# Ethyl Acetate Fraction of Chestnut Honey Attenuates Scopolamine-Induced Cognitive Impairment in Mice and Glutamate-Induced Neurotoxicity in HT22 Cells

**DOI:** 10.3390/antiox13111346

**Published:** 2024-11-02

**Authors:** Yun Hee Jeong, Wei Li, Hye Jin Yang, Se-Gun Kim, Hong Min Choi, Jang-Gi Choi, You-Chang Oh

**Affiliations:** 1 Korean Medicine (KM)-Application Center, Korea Institute of Oriental Medicine, 70, Cheomdanro, Dong-gu, Daegu 41062, Republic of Korea; runxi0333@kiom.re.kr (Y.H.J.); liwei1986@kiom.re.kr (W.L.); hjyang@kiom.re.kr (H.J.Y.); 2 Department of Agricultural Biology, National Institute of Agricultural Sciences, Rural Development Administration, Wanju 55365, Republic of Korea; kimsegun@korea.kr (S.-G.K.); sgiantm@korea.kr (H.M.C.)

**Keywords:** ethyl acetate fraction of chestnut honey, cognitive impairment, neuroprotective effect, antioxidant, neurodegenerative diseases, nuclear factor-E2-related factor 2

## Abstract

Chestnut honey has various benefits, such as antioxidative, anti-inflammatory, immunomodulatory, antibacterial, and antiviral effects. However, the effects of chestnut honey or the ethyl acetate fraction of chestnut honey (EACH) on neurodegenerative diseases and their related cognitive impairment and neurotoxicity have not yet been established. Therefore, in this study, we investigated the mitigating effect of the EACH on scopolamine (SCO)-injected cognitive decline in mice and glutamate-exposed neurotoxicity in HT22 cells. EACH administration significantly reversed SCO-induced cognitive decline in mice, as demonstrated through the Morris water maze and passive avoidance tests. The EACH treatment showed a significant alleviation effect by recovering more than 80% of the cell viability decrease induced by glutamate exposure in the HT22 neuronal cell model. Furthermore, the EACH significantly reduced reactive oxygen species accumulation, lactate dehydrogenase release, mitochondrial depolarization, and neuronal apoptosis. The EACH regulated the level of apoptosis-related proteins, induced the nuclear translocation of nuclear factor-E2-related factor 2 (Nrf-2) and the expression of related antioxidant proteins, and induced the phosphorylation of tropomyosin-related kinase receptor B (TrkB)/cAMP-calcium response element-binding protein (CREB) and the expression of brain-derived neurotrophic factor. These data indicate that the EACH can prevent neurons from oxidative damage and improve cognitive dysfunction by activating Nrf-2 and TrkB/CREB signaling pathways. Therefore, the EACH demonstrates potential therapeutic value in mitigating oxidative stress-induced neurotoxicity, cognitive decline, and related neurodegenerative diseases.

## 1. Introduction

Chestnut honey (CH) is collected from the flowers of the chestnut tree (*Castanea sativa*). It is dark brown in color with a bitter taste and strong aroma [1]. CH contains sugars, minerals, vitamins, proteins, enzymes, and amino acids. It is rich in phenolic acids and flavonoids and exhibits the highest antioxidant activity among the various types of honey [2,3]. Previous research has confirmed the anti-inflammatory, immunomodulatory, antibacterial, and antiviral effects of CH [4,5,6,7]. However, its effects on cognitive function and neuroprotection have not been investigated.

Neurodegenerative diseases are a group of irreversible neurological disorders characterized by neuronal dysfunction and progressive degeneration in multiple regions of the brain, and although the exact etiology is not yet known, a complex interaction between genetic and environmental factors has been suggested [8,9]. Among them, Alzheimer’s disease (AD) is a neurodegenerative disorder characterized by the progressive loss of cognitive function, especially learning and memory, as indicated by such early symptoms as short-term memory impairment [10,11]. AD is the most common form of dementia, accounting for 60–70% of all neurodegenerative diseases [12]. Its impact is expected to reach epidemic levels due to the increasing life expectancy worldwide and the current lack of effective treatments [13]. The scopolamine (SCO)-induced cognitive impairment model, which mimics that in AD, is one of the most efficient methods of studying the cognitive enhancement properties of drugs since SCO induction mimics both the behavioral and molecular features of AD [14,15]. SCO-induced amyloid beta (Aβ) plaques inhibit choline acetyltransferase activity, thereby hindering acetylcholine (ACh) synthesis and release and reducing choline uptake, which results in cognitive impairment similar to that in human AD [16].

Oxidative stress and excitotoxic dysfunction are contributors to neuronal cell damage and synaptic dysfunction, which are the main features of AD [17]. Glutamate is a primary mediator of the excitatory neurotransmitter for a variety of normal brain functions, where it plays a crucial role in neuronal differentiation, migration, and survival during brain development [18]. However, an abnormal increase in glutamate secretion can cause neuronal cell death due to oxidative stress or excitotoxicity [19,20]. Excessive glutamate concentrations increase the reactive oxygen species (ROS) level, which can lead to damage to proteins, DNA, and lipid membranes, ultimately leading to mitochondrial dysfunction and cell damage [21,22]. Therefore, regulating ROS production alleviates the risk of oxidative stress and is thus an effective method for treating or preventing neurodegenerative diseases.

The essential cellular transcription factor Nuclear factor-E2-related factor 2 (Nrf-2) promotes the secretion of several antioxidant enzymes in the CNS, such as NAD(P)H quinone oxidoreductase 1 (NQO1), glutamate–cysteine ligase catalytic subunit (GCLC), and heme oxygenase (HO)-1 [23,24,25]. Numerous studies have demonstrated that the Nrf-2/antioxidant response element (ARE) signaling pathway protects neurons from oxidative damage [26,27], and its activation exerts neuroprotective effects against Aβ toxicity and improves cognitive function in transgenic AD mice [28]. Another neuroprotective mechanism is closely correlated with the production of brain-derived neurotrophic factor (BDNF) that is associated with neuronal proliferation, differentiation, growth, synaptic plasticity, and cognitive function [29,30]. BDNF binds to tropomyosin-related kinase receptor B (TrkB) and initiates multiple signaling cascades [27,28,29]. In turn, BDNF is reported to activate cAMP-calcium response element-binding protein (CREB), a transcription factor related to long-term memory formation [30]. Therefore, the activation of the NRF2/ARE and TrkB/CREB/BDNF signaling pathways may be an important therapeutic target for neurodegenerative disorders.

In the current study, we measured the ameliorative activities of the ethyl acetate fraction of chestnut honey (EACH) on SCO-induced cognitive impairment in mice. We investigated the changes in learning ability, including spatial memory and working memory, of mice administered the EACH through behavioral tests. In addition, we investigated the neuroprotective effect of EACH and its underlying molecular mechanism in HT22 hippocampal cells exposed to glutamate. Also, we identified the major components of the EACH using ultrahigh-performance liquid chromatography (UHPLC)-Q-Exactive Orbitrap MS analysis.

## 2. Materials and Methods

### 2.1. Preparation of Ethyl Acetate Fraction of Chestnut Honey (EACH)

CH from *Castanea crenata* (10 kg) was extracted with ethyl acetate (20 L) at room temperature (RT) using an overhead stirrer (DAIHAN Scientific Co., Wonju, Republic of Korea) at 200 rpm for 1 h. This process was repeated three times. The combined supernatant was concentrated in vacuo, yielding ethyl acetate extract of chestnut honey (29.2 g).

### 2.2. UHPLC-Q-Exactive Orbitrap MS Analysis

To identify the phytochemicals in the EACH, MS analysis was conducted using a Thermo Dionex Ultimate 3000 system, coupled with a Thermo Q-Exactive Orbitrap mass spectrometer (Thermo Fisher Scientific, San Jose, CA, USA) equipped with a heated electrospray ionization (HESI) interface. Detailed analytical conditions and methods are described in previous studies [31,32]. Data acquisition and analysis were carried out using Xcalibur v.4.2 software (Thermo Fisher Scientific, Foster, CA, USA). Methanol, acetonitrile, water, and formic acid used in the analysis were all MS-grade and purchased from Thermo Fisher Scientific (Pittsburgh, PA, USA).

### 2.3. Animals and Treatment Ddosage

C57BL/6 mice (4 weeks old) were acquired from Samtako BioKorea (Osan, Republic of Korea). All animal experiments were approved by the guidelines for the Animal Care and Use Committee of the Korea Institute of Oriental Medicine (Reference number #23-096). In addition, according to the 3Rs principles, the number of mice per group was set to 8, which was considered the minimum, and the mice breeding environment was maintained to be clean and less exposed to stress. Also, the animal experiments were conducted under the principle that if the mouse showed excessive weight loss, inability to walk, or other serious abnormal symptoms during the breeding and experiment process, the experiment would be stopped immediately, and the mouse would be euthanized. Mice were housed at four heads per cage under automatic temperature control and a 12 h light–dark cycle. The mice were fed a standard laboratory diet and water ad libitum. The mice were then randomly divided into four groups (*n* = 8; Normal, SCO, SCO + EACH 100 mg/kg, and SCO + EACH 300 mg/kg) and were acclimatized to the facility for 7 days before the experiments. All mice were administered orally with saline (Normal and SCO groups) or EACH (EACH 100 and 300 mg/kg groups) using a zonde once daily for four weeks, and 1 mg/kg SCO was administered intraperitoneally once daily for the last week.

### 2.4. Morris Water Maze and Probe Test

The Morris water maze (MWM) test is a behavioral test designed to evaluate the spatial memory function of mice. A rubber pool was filled with approximately 70% water with the water at a temperature of 22–24 °C. The MWM pool was filled with purified water diluted with non-toxic white paint to hide the platform from the mice. Each trial was conducted for 1 min, during which the mice were allowed to find and climb onto the hidden platform. The experiment was terminated if the mouse failed to reach the platform within 1 min. From days 1 to 5 of the experiment, all mice learned the location of the platform and the orientation around it, regardless of whether they reached the platform. Two days after the end of the exploration test, the platform in the northeastern (NE) quadrant of the rubber pool was removed, and each mouse performed a probe test in which they were allowed to swim for the same amount of time. These behavioral tests were performed as described previously [33].

### 2.5. Passive Avoidance Test

The passive avoidance (PA) test was performed to assess the working memory of the mice. The apparatus was trough-shape and consisted of two rooms of equal area, an illuminated chamber and a darkened chamber, which were connected to each other by an automatic door. The mice started the test in the lit chamber and were shocked through an electric grid floor when they moved into the darkened chamber. The latency into the darkened chamber and time were recorded up to 3 min. The experiment was terminated if the mice did not move into the dark chamber within the set time. This test was performed as described previously [34].

### 2.6. HT22 Neuronal Cell Culture, Stimulation, and Drug Treatment

The HT22 mouse hippocampal neuronal cell line was provided by Dr. Younghoon Go of the Korea Institute of Oriental Medicine (Daegu, Republic of Korea). The initial passage of frozen HT22 cells was 10, and cells within 5–6 subcultures after thawing were used in the experiments. Cells were grown in Dulbecco’s Modified Eagle Medium (DMEM) medium containing 10% fetal bovine serum (FBS) and 1% penicillin/streptomycin antibiotics at 37 °C in 5% CO_2_. DMEM, FBS, and antibiotics were acquired from HyClone (Logan, UT, USA). The medium was replaced daily, and the cells were subcultured every two days at an appropriate rate. After two days of subculture, when the cells reached 80–90% confluency, they were used for subsequent experiments. To induce neuronal damage, the culture medium was changed to fresh DMEM and glutamate of 5 mM was added in the presence or absence of the EACH (250, 500, or 750 μg/mL).

### 2.7. Cell Viability Assay

To determine the cytotoxicity of the EACH, HT22 cells were plated into 96-well culture plates and incubated with the EACH or 5 mM glutamate for 24 h. For the cell viability test, the cells were treated with a cell counting kit (CCK) solution and incubated for an additional 1 h. The CCK solution was purchased from Dojindo Molecular Technologies (Kumamoto, Japan). Cell viability was detected at 450 nm optical density using an ELISA reader (SpectraMax i3, Molecular Devices, San Jose, CA, USA).

### 2.8. Lactate Dehydrogenase (LDH) Release Assay

The release of lactate dehydrogenase (LDH) in the culture medium was assessed with an LDH assay kit according to the instruction manual. The LDH assay kit was obtained from Abcam (Cambridge, UK). The HT22 cells were pretreated with the EACH and added 5 mM glutamate for 24 h, and the cells were centrifuged on the microplate at 250 g for 10 min. After 10 µL, the supernatant was transferred into a 96-well culture plate, and 100 μL of freshly prepared LDH reaction mixture was added to each well and incubated for up to 30 min at RT. LDH activity was determined by an ELISA reader at 450 nm.

### 2.9. Determination of Intracellular Reactive Oxygen Species (ROS) Accumulation

Intracellular ROS accumulation was measured using the 2,7-Dichlorohydrofluorescein diacetate (H_2_DCFDA) fluorometric assay, as described previously [35]. H_2_DCFDA was purchased from Invitrogen (Carlsbad, CA, USA). The HT22 cells were pretreated with the EACH and then stimulated with 5 mM glutamate for 6 h. The cells were washed twice with phosphate-buffered saline (PBS) and then incubated with a 15 μM H_2_DCFDA probe for 25 min at 37 °C in the dark. The signal of H_2_DCFDA was analyzed by a fluorescence microplate reader at an excitation wavelength of 488 nm and emission wavelength of 525 nm. Fluorescence images were visualized by a fluorescence microscope (Eclipse Ti, Nikon, Tokyo, Japan).

### 2.10. JC-1 Staining for Analysis of Mitochondrial Membrane Potential (MMP)

The fluorescent probe JC-1 was used to determine the mitochondrial membrane potential (MMP) of the HT22 cells after the EACH and glutamate treatment. JC-1, a fluorescent probe, was purchased from Biotium (Hayward, CA, USA). The cells were suspended in JC-1 solution for 20 min in a humidified incubator at 37 °C and then washed 2 times with PBS. JC-1 fluorescence was determined by ELISA reader excitation/emission wavelengths at 490/530 nm for green (monomer form) and 520/590 nm for red (aggregate form) fluorescence. Representative fluorescence images were obtained using a fluorescence microscope.

### 2.11. Apoptotic Cell Death Assessment by Flow Cytometry

HT22 cell apoptosis was detected by flow cytometry. The FITC–annexin V staining kit was used following the manufacturer’s instructions. The annexin V–fluorescein isothiocyanate (FITC)/propidium iodide (PI) apoptosis detection kit was acquired from BD Biosciences (Franklin Lakes, NJ, USA). The cells were pretreated with the EACH and stimulated 5 mM glutamate for 24 h. Then, the cells were washed 2 times with PBS and centrifuged at 13,000 rpm, and PI and FITC-conjugated annexin V were added at RT in the darkness for 20 min. The cells were measured using a FACS Calibur system (BD Biosciences).

### 2.12. Preparation of Whole Cell and Nuclear Lysates

Cell lysates were extracted using RIPA buffer with a protease and phosphatase inhibitor cocktail (Roche, Basel, Switzerland). Nuclear fractions were isolated using a NE-PER™ nuclear and cytoplasmic extraction reagent (Thermo Fisher Scientific) according to the instruction manual.

### 2.13. Western Blotting

The lysate obtained from HT22 cells was normalized using a bicinchoninic acid kit (Thermo Fisher Scientific) and denatured at 95 °C for 5 min. The same amounts of protein were resolved by 8–15% sodium dodecyl sulfate–polyacrylamide gel electrophoresis for 3 h and transferred onto polyvinylidene fluoride (PVDF) membranes. The PVDF membrane was sealed with 3% BSA for 1 h at RT. Then, the PVDF membrane was cultured in 3% BSA at 4 °C overnight with a 1:1000 dilution of each primary antibody. The PVDF membranes were then cultured with HRP-conjugated secondary antibodies in 1:500 for 1 h and analyzed by enhanced chemiluminescence by a ChemiDoc^TM^ Touch Imaging System (Bio-Rad, Hercules, CA, USA). Primary and secondary antibodies were acquired from Cell Signaling Technology (Boston, MA, USA) and Santa Cruz Biotechnology (Santa Cruz, CA, USA). Each protein band was normalized to β-actin or Lamin B1 and quantified using Image J software (Version 1.53e, National Institutes of Health, Bethesda, MD, USA). The primary and secondary antibodies used are listed in Table 1.

### 2.14. Statistical Analysis

The data are shown as the mean ± standard error of the mean. Statistical significance was determined by a one-way analysis of variance followed by Dunnett’s test after comparing each treatment group vs. SCO or glutamate. Statistical analyses were performed using GraphPad prism 8.0 software, and ^#^ *p* < 0.05 (vs. normal or control), * *p* < 0.05, ** *p* < 0.01, and ^†^ *p* < 0.001 (vs. SCO or glutamate) were considered as statistically significant differences.

## 3. Results

### 3.1. EACH Alleviated Cognitive Impairment by Scopolamine (SCO) Injection

**Figure 1:**
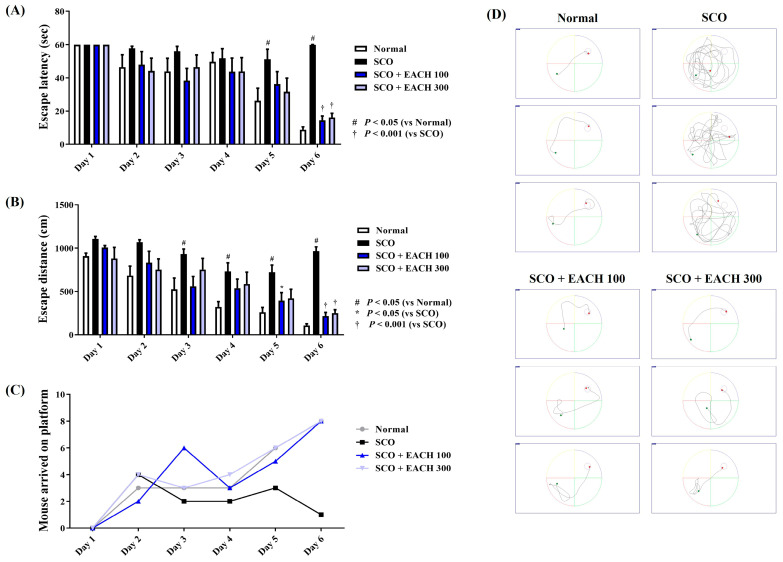
Effects of ethyl acetate fraction of chestnut honey on scopolamine (SCO)-induced mice cognitive impairment in the Morris water maze (MWM) test. Spatial memory function in mice was determined by (**A**) escape latency, (**B**) escape distance, (**C**) number of mice arriving at the platform, and (**D**) swimming trajectory in the MWM test. (**A**–**C**) Graphs show the daily MWM test results for 6 days. (**D**) Images represent the swimming trajectory of mice in each group on Day 6. Data are expressed as mean ± standard error of the mean (*n* = 8). ^#^ *p* < 0.05 (vs. normal), * *p* < 0.05, and ^†^ *p* < 0.001 (vs. SCO).

We carried out the MWM test to examine the effects of the EACH on SCO-induced decreases in spatial memory function and learning ability. During the 6 days of training and testing, normal mice showed relatively quick learning and good record shortening, whereas the mice given only SCO showed poor learning and memory. Compared with the SCO group, the average escape distance and latency of the mice administered with EACH were significantly reduced. In particular, the escape latency on day 6 and the escape distance on days 5 and 6 were statistically significantly shortened (Figure 1A,B). In addition, the number of mice that reached the platform within 60 s and successfully escaped the water maze also increased steeply in the EACH group over time (Figure 1C). The differences in mice cognitive performance between the groups were also clearly demonstrated by the swimming trajectories on day 6 (Figure 1D).

### 3.2. EACH Administration Effectively Suppressed SCO-Induced Memory Loss

**Figure 2:**
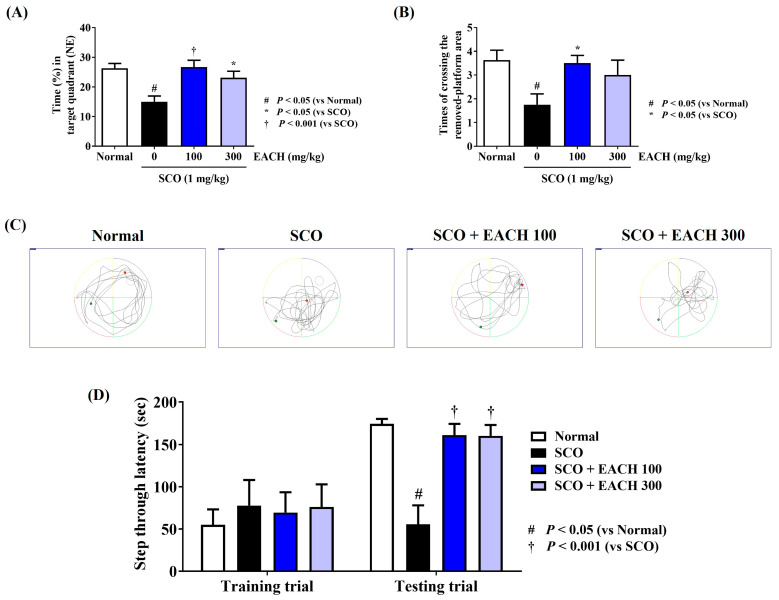
Effects of ethyl acetate fraction of chestnut honey on mice memory loss induced by scopolamine (SCO) injection, as assessed using (**A**–**C**) probe and (**D**) passive avoidance (PA) tests. (**A**) Time spent in the northeast quadrant, (**B**) times the mice crossed the former platform location and (**C**) swimming trajectory in the probe test. (**D**) Step-through latency of the training and test trials in the PA test. (**C**) Images represent the swimming trajectory of mice in each group on the probe test. Data are expressed as mean ± standard error of the mean (*n* = 8). ^#^ *p* < 0.05 (vs. normal), * *p* < 0.05, and ^†^ *p* < 0.001 (vs. SCO).

The probe test was performed to analyze the time the mice spent in the NE quadrant, which was the original platform location, and the number of times they passed the location of the removed platform. Compared with the normal group, the SCO-administered group spent less time in the NE quadrant, and the number of times they passed the former platform location was also lower. In contrast, the group that was administered with EACH spent more time in the NE quadrant, and the number of times they crossed the platform location was also significantly higher than that in the SCO group (Figure 2A–C). However, the 100 mg/kg EACH group showed statistically significant efficacy in both parameters, but the 300 mg/kg EACH group did not, so no dose dependency was observed. To evaluate the working memory of the mice in each group, the PA test was performed 2 days after the probe test, and the test trial was conducted 1 day after the training trial. The EACH group showed a statistically significant increase in the average step-through latency compared with the SCO group at both the 100 and 300 mg/kg doses (Figure 2D).

### 3.3. EACH Restored the Decreased Cell Viability and Effectively Reduced LDH Leakage in HT22 Neurons Exposed to Glutamate

**Figure 3:**
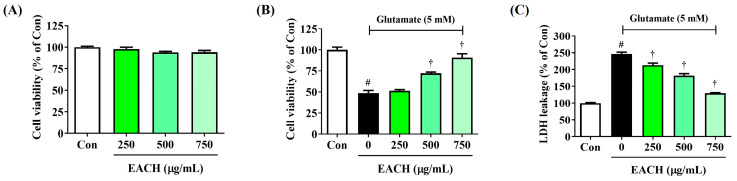
Effects of ethyl acetate fraction of chestnut honey (EACH) on glutamate-induced cytotoxicity in HT22 cells. (**A**) Cell viability when treated with EACH alone, (**B**) the effect of EACH on the decrease in cell viability induced by glutamate, and (**C**) the effect of EACH on the increase in lactate dehydrogenase leakage induced by glutamate. HT22 cells were incubated with EACH or glutamate for 24 h. Data are expressed as mean ± standard error of the mean of three independent experiments. ^#^ *p* < 0.05 (vs. control) and ^†^ *p* < 0.001 (vs. glutamate).

We tested the neuroprotective effects of the EACH in neuron cells exposed to the neurotoxic glutamate to cross-validate the ameliorative effects of the EACH on cognitive impairment demonstrated in the mouse model experiment. In the cell viability test using CCK solution, the EACH treatment alone did not affect cell viability up to 750 μg/mL when treated for 24 h, indicating that the EACH was not toxic to HT22 neurons at that concentration (Figure 3A). When glutamate was administered to HT22 cells, an apoptosis rate of approximately 50% was observed. In contrast, pretreatment with the EACH significantly inhibited cell death in a concentration-dependent way (Figure 3B). Furthermore, LDH leakage, which increased by approximately 150% with glutamate exposure, was concentration-dependently inhibited by EACH pretreatment (Figure 3C). Specifically, the inhibitory effects on cell death and LDH leakage were statistically significant when the EACH was treated at 500 μg/mL or higher.

### 3.4. EACH Inhibited Excessive Intracellular ROS Production Induced by Glutamate

**Figure 4:**
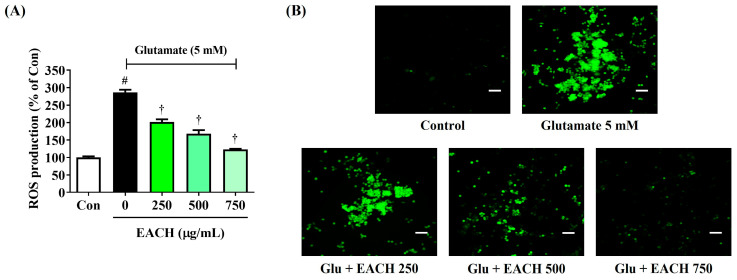
Effects of ethyl acetate fraction of chestnut honey (EACH) on the production of intracellular reactive oxygen species (ROS) in glutamate-exposed HT22 cells. Cells were incubated with glutamate in the presence or absence of EACH. ROS production was assessed using a (**A**) fluorescence microplate reader and (**B**) fluorescence microscope. (**B**) Images represent the three independent experiments; scale bar = 200 μm. Data are expressed as mean ± standard error of the mean. ^#^ *p* < 0.05 (vs. control) and ^†^ *p* < 0.001 (vs. glutamate).

We also examined the effect of the EACH on the accumulation of ROS induced by glutamate treatment using the H_2_DCFDA fluorescence assay. Glutamate exposure rapidly increased intracellular ROS levels, which were reduced by the EACH pretreatment in a concentration-dependent way with statistical significance, as shown in the fluorescence microscopy (Figure 4). These findings show that the EACH can suppress excessive ROS production in neurons.

### 3.5. EACH Restored Decreased MMP in Neurons Caused by Glutamate Exposure

**Figure 5:**
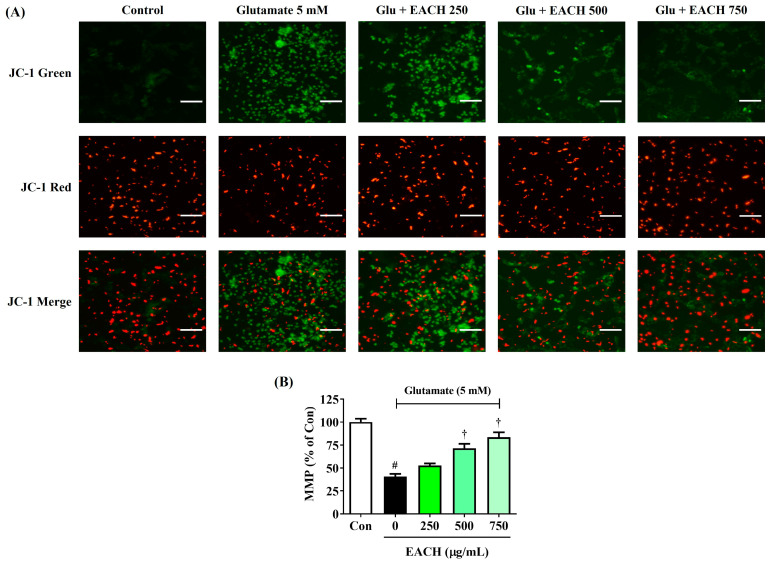
Effects of ethyl acetate fraction of chestnut honey on mitochondrial dysfunction in glutamate-exposed HT22 cells. (**A**) Mitochondrial membrane potential was assessed via fluorescence microscopy using JC-1 staining. (**A**) Images represent the three independent experiments; scale bar = 100 μm. (**B**) The histogram shows the red/green fluorescence intensity ratio. Data are expressed as mean ± standard error of the mean. ^#^ *p* < 0.05 (vs. control) and ^†^ *p* < 0.001 (vs. glutamate).

To determine whether the neuroprotective effect of EACH was due to the prevention of mitochondrial dysfunction in neurons, we examined the changes in MMP using JC-1 fluorescence staining. JC-1 accumulates in the matrix of healthy mitochondria (red fluorescence) and appears in a monomeric form (green fluorescence) when mitochondria are depolarized during cell death after exposure to toxic agents. Healthy control cells showed high red fluorescence and low green fluorescence (Figure 5). However, glutamate exposure rapidly changed the MMP of neurons, significantly increasing the green fluorescence intensity while significantly decreasing the red fluorescence intensity, the latter of which indicated MMP loss. On the other side, the EACH treatment prevented glutamate-induced MMP loss, as evidenced by decreased green fluorescence and increased red fluorescence (Figure 5). EACH, treated at concentrations of 500 and 750 μg/mL, showed significant recovery of MMP in a concentration-dependent manner, reaching statistical significance.

### 3.6. EACH Treatment Inhibited Neuronal Cell Death Caused by Glutamate Exposure

**Figure 6:**
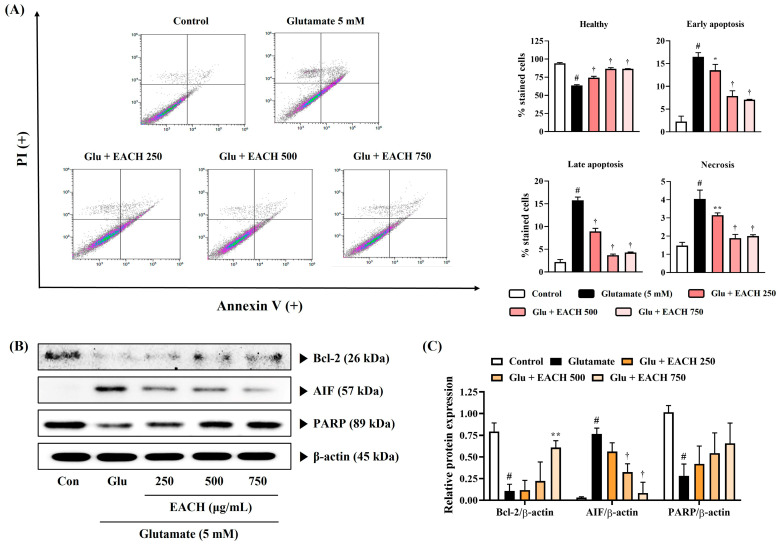
Effects of ethyl acetate fraction of chestnut honey on apoptosis in glutamate-exposed HT22 cells. (**A**) HT22 cell apoptosis was evaluated using flow cytometry. (**B**,**C**) The expression levels of apoptosis-related proteins were determined by Western blotting. (**B**) Blot images represent the three independent experiments. (**C**) The histograms show protein expression levels relative to β-actin. Data are expressed as mean ± standard error of the mean. ^#^ *p* < 0.05 (vs. control), * *p* < 0.05, ** *p* < 0.01, and ^†^ *p* < 0.001 (vs. glutamate).

To determine whether the EACH attenuated glutamate-induced neuronal cell death, flow cytometry using annexin V-PI double staining was carried out. As demonstrated by flow cytometry, upon glutamate exposure for 24 h, apoptosis among HT22 cells increased significantly compared with the untreated control group (Figure 6A). In contrast, the EACH attenuated glutamate-induced neuronal cell death in a concentration-dependent way with statistical significance (Figure 6A). Moreover, this effect of EACH was significant during early and late apoptosis and necrosis.

### 3.7. Regulatory Effect of EACH on Apoptosis-Related Proteins Expression in HT22 Neurons

To determine the protein changes associated with the antiapoptotic effect of EACH, we evaluated the effect of EACH on the expression of several related proteins in the HT22 cells exposed to glutamate. As shown on Western blotting (Figure 6B,C), glutamate exposure increased the expression of the apoptotic protein apoptosis-inducing factor (AIF) and decreased the expression of the antiapoptotic proteins B-cell lymphoma 2 (Bcl-2) and poly (ADP-ribose) polymerase (PARP) in HT22 cells. In contrast, the EACH pretreatment decreased AIF expression and restored Bcl-2 and PARP expression (Figure 6B,C). In particular, the effects of the EACH on Bcl-2 and AIF expression were significant and concentration-dependent. However, PARP expression showed a pattern of restoration by the EACH pretreatment, but statistical significance was not observed. The flow cytometry and Western blotting results indicate that the neuroprotective effect of the EACH is partly influenced by the reduction in apoptosis.

### 3.8. EACH Induced the Activation of Nuclear Factor-E2-Related Factor 2 and the Expression of Antioxidant Enzymes in HT22 Neurons Exposed to Glutamate

**Figure 7:**
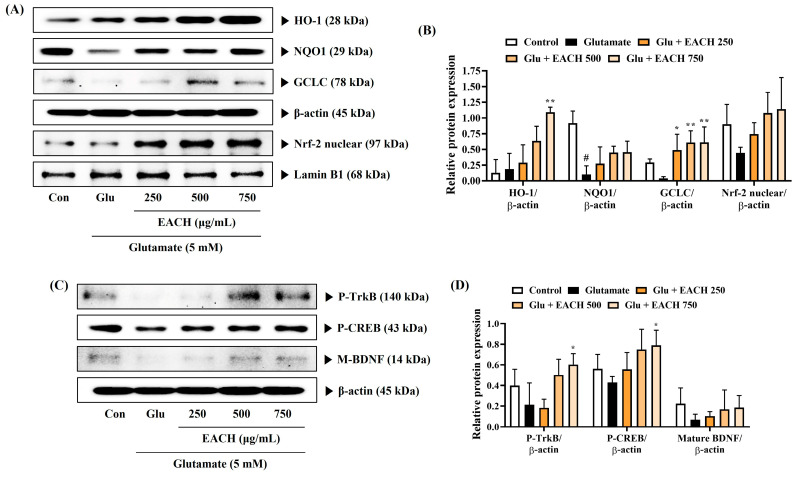
Effects of ethyl acetate fraction of chestnut honey on the activation of (**A**,**B**) nuclear factor-E2-related factor 2 and associated antioxidant enzymes and (**C**,**D**) tropomyosin-related kinase receptor B/cAMP-calcium response element-binding protein/brain-derived neurotrophic factor pathway in glutamate-exposed HT22 cells. (**A**–**D**) The expression levels of all proteins were determined by Western blotting. (**A**,**C**) Blot images represent the three independent experiments. (**B**,**D**) The histograms show protein expression levels relative to β-actin or Lamin B1. Data are expressed as mean ± standard error of the mean. ^#^ *p* < 0.05 (vs. control), * *p* < 0.05, and ** *p* < 0.01 (vs. glutamate).

In various neurodegenerative disease models, Nrf-2 activation induces antioxidant enzyme expression, which inhibits oxidative stress and exerts neuroprotective effects [36]. Therefore, we analyzed the effects of the EACH on Nrf-2 translocation into the nucleus and the expression of antioxidant enzymes in HT22 cells to investigate the relationship between the neuroprotective effect of the EACH and Nrf-2 activation and the expression of related antioxidant enzymes. The EACH pretreatment statistically significantly induced the expression of HO-1 and GCLC and also slightly increased NQO1 expression (Figure 7A,B). Furthermore, the nuclear translocation of Nrf-2 showed a tendency to be increased by the EACH treatment, although this was not statistically significant (Figure 7A,B). Therefore, the EACH induced the expression of several antioxidant enzymes through Nrf-2 activation, which signifies that the antioxidant properties of the EACH contribute somewhat to its neuroprotective activity.

### 3.9. EACH Increased Mature Brain-Derived Neurotrophic Factor Expression Through the Activation of Tropomyosin-Related Kinase Receptor B/cAMP-Calcium Response Element-Binding Protein Pathway

Because the TrkB/CREB/BDNF pathway is involved in the growth and survival of neuronal cells, we investigated how the EACH affected BDNF expression and TrkB/CREB pathway activation. EACH more effectively activated TrkB and CREB by inducing phosphorylation compared with the glutamate treatment alone (Figure 7C,D). Furthermore, the EACH showed a pattern of inducing mature BDNF expression, although it was not statistically significant (Figure 7C,D), suggesting that the EACH promoted BDNF expression related to neuronal growth and survival by TrkB/CREB pathway activation. Therefore, the neuroprotective effect of the EACH may be partially related to the activation of the TrkB/CREB/BDNF signaling pathway.

### 3.10. Qualitative Analysis of EACH

We performed a qualitative analysis using UHPLC-Q-Exactive Orbitrap MS to confirm the presence of phytochemicals in the EACH. Chromatographic separation was achieved using an Acquity BEH C_18_ analytical column (100 × 2.1 mm, 1.7 μm, Waters, Milford, MA, USA) with gradient elution with 0.1% (*v*/*v*) formic acid in water (solvent A) and acetonitrile (solvent B), maintained at 40 °C for optimal separation. To ensure accuracy in the MS spectrum acquisition and molecular mass measurements of the analyte, MS analysis was performed in both positive and negative ionization modes using the HESI source. The MS spectra were acquired at a normalized collision energy of 25 eV in full MS and ddMS^2^ scan modes over a scan range of 100–1500 *m*/*z*.

**Figure 8:**
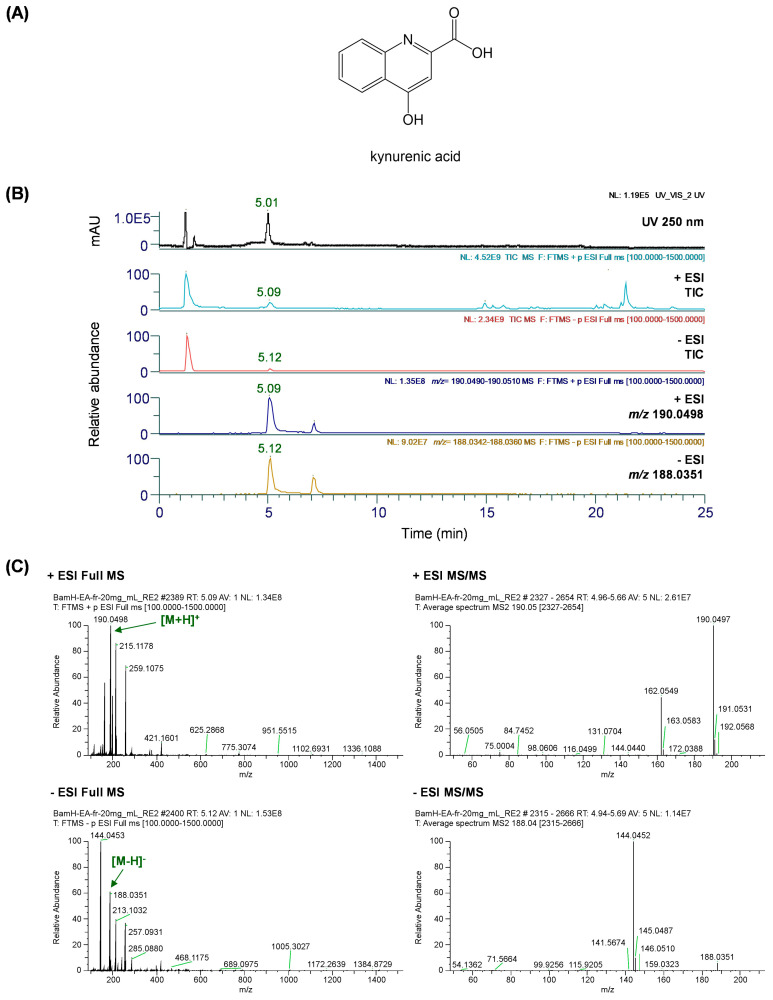
UHPLC-Q-Exactive Orbitrap MS analysis of ethyl acetate fraction of chestnut honey. (**A**) Chemical structures of kynurenic acid. (**B**) UV chromatogram at wavelengths of 250 nm, the total ion chromatograms, along with the extracted ion chromatograms for the precursor ion *m*/*z* values of the analyte. (**C**) Full MS and MS2 spectra of the analyte in both positive and negative ionization modes.

As a result of the analysis, the quinoline alkaloid kynurenic acid (KYNA) was identified in the EACH. KYNA is one of the tryptophan catabolites formed via the kynurenine pathway, and according to previously reported research literature, it is known as a marker compound mainly present in chestnut honey [4,37]. Figure 8A shows the chemical structures of KYNA. Figure 8B presents the UV and the total ion chromatograms (TIC), along with the extracted ion chromatograms (EIC) for the precursor ion *m*/*z* values of the analyte. Figure 8C shows the full MS and MS2 spectra of the analyte in both positive and negative ionization modes.

In the positive ionization mode, KYNA confirmed at retention time (*t*_R_) of 5.09 min presented precursor ions at *m*/*z* 190.0498 [M+H]^+^ (calcd. 190.0499), corresponding to the molecular formula C_10_H_7_NO_3_, as provided by Orbitrap. The fragment ions shown in the MS/MS spectrum were *m*/*z* 190.0497 [M+H]^+^ and 162.0549 [M-C=O+H]^+^. Similarly, KYNA was identified in the negative ionization mode. Detected at 5.12 min, KYNA showed a precursor ion at *m*/*z* 188.0351 [M-H]^−^ (calcd. 188.0353), corresponding to the molecular formula C_15_H_12_O_7_ provided by Orbitrap. Additionally, the fragment ion of the MS/MS spectrum was *m*/*z* 144.0452 [M-COOH]^−^. Comparing these results with previous literature, this compound was identified as KYNA [38,39,40]. Based on the area calculations of the positive and negative mass spectra of KYNA, we determined that the content of KYNA in the EACH is 69.97 mg/g. Additionally, the KYNA concentrations in 100 mg/kg and 300 mg/kg EACH that were administered to mice were 6.997 mg/kg and 20.991 mg/kg, respectively.

## 4. Discussion

Although CH exerts various physiological activities, its efficacy in treating neurodegenerative diseases has not been studied. We researched the beneficial effects of the EACH on SCO-induced mouse cognitive impairment and glutamate-induced neurotoxicity. SCO is a tropane alkaloid that interferes with learning and short-term memory by blocking the effects of ACh in the CNS [41]. The SCO-induced cognitive impairment mouse model has been widely used as a standard experimental model for cognitive and memory disorders [14,15,16]. We performed behavioral tests, including MWM, probe, and PA tests, using SCO-induced amnesic mice to determine whether the EACH improved cognitive function. Mice administered with the EACH showed a significant decrease in escape distance and latency in finding the platform compared with the SCO group. The EACH group had a higher rate of successful escape from the water maze within 60 s. In addition, mice administered with the EACH showed a higher average step-through latency in the PA test compared with the SCO group. Thus, EACH administration effectively alleviated the decline in spatial memory function and working memory in mice induced by SCO.

Oxidative stress and excitotoxic dysfunction are closely involved in the pathological processes of various neurodegenerative diseases [42]. They are implicated in early AD and cause mitochondrial dysfunction in neurons [17,43]. In addition, high glutamate levels in the brain induce excessive ROS accumulation and neuronal damage, which feature in the pathophysiology of AD [19,20,21]. Therefore, reducing oxidative stress and neuronal cell death caused by high glutamate levels may be a promising strategy for the prevention or treatment of neurodegenerative diseases. Thus, the EACH emerges as a novel candidate for the regulation of excitotoxicity-related neurodegenerative diseases. The EACH pretreatment significantly increased cell viability and decreased LDH leakage and neuronal apoptosis in the HT22 cells exposed to glutamate. Furthermore, the EACH significantly inhibited glutamate-induced ROS overproduction and improved MMP reduction in a concentration-dependent manner. These results demonstrate that the EACH promotes neuronal survival by inhibiting ROS accumulation in neurons and restoring mitochondrial function.

Activation of the Nrf-2/ARE pathway exerts protective effects against neuronal apoptosis, thereby reducing ROS and lipid peroxidation and improving mitochondrial dysfunction [44,45]. Nrf-2 is an important transcription factor that regulates cellular redox homeostasis and acts as an important protector of the antioxidant response system [45]. In normal cells, Nrf-2 exists in the cytoplasm in an inactive form, bound to Kelch-like ECH-associated protein 1 (Keap1) [45]. However, upon specific stimulation, the Nrf-2/Keap1 complex is dissociated, and Nrf-2 translocates to the nucleus and binds to ARE to stimulate antioxidant enzyme production in neurons [46]. Therefore, to determine the value of the EACH as a preventive or therapeutic agent for neurodegenerative diseases [47,48], we explored how the EACH affected the Nrf-2/ARE signaling pathway and the expression of antioxidant enzymes. Western blotting revealed that the EACH pretreatment effectively increased Nrf-2 nuclear translocation and antioxidant enzyme expression, including HO-1, NQO1, and GCLC. This evidence confirms that the neuroprotective effect of the EACH is initiated by activating the Nrf-2/ARE signaling pathway.

The effects of the EACH on the activation of the TrkB/CREB signaling pathway and BDNF production were also examined. BDNF is a major neurotrophic factor that activates its receptor TrkB and is closely related to hippocampal-dependent learning and memory functions [30]. Upon BDNF stimulation, TrkB activates CREB to promote the synaptic plasticity of neurons, thereby enhancing learning and memory processes [49]. Recent studies have shown that the TrkB/CREB/BDNF signaling pathway protects against oxidative stress-induced neuronal death and activates antioxidative responses to enhance learning and cognitive abilities [50,51]. As demonstrated by the experimental results, the EACH pretreatment significantly increased the phosphorylation of both TrkB and CREB and induced mature BDNF production. Thus, the EACH can attenuate glutamate-induced neurotoxicity by activating the TrkB/CREB pathway and inducing the expression of BDNF in HT22 cells.

Next, we performed phytochemical analysis using UHPLC-Q-Exactive Orbitrap MS to investigate the relationship between the bioactivity of the EACH and its components. As a result, KYNA was identified as the main component of the EACH. Previous studies have demonstrated that changes in central and peripheral KYNA synthesis are involved in the pathogenesis of several neurodegenerative diseases, and KYNA deficiency has been shown to partially contribute to the pathogenesis of neuronal loss [52]. KYNA has been shown to modulate neurotransmission and exert noncompetitive antagonistic effects at both glutamatergic and nicotinergic neurotransmission, thereby modulating α7-nicotinic acetylcholine receptors and exerting protective effects against glutamate-induced excitotoxicity [53,54]. Another study showed that KYNA could be a potential endogenous antioxidant by scavenging ROS in a manner independent of N-methyl-D-aspartic acid and nicotinic receptors [55]. In addition, it was demonstrated that increasing blood KYNA levels reduces extracellular glutamate in the brain, prevents spatial memory deficits and synapse loss in an AD transgenic mouse model, and reduces microglial activation [56]. Therefore, the ameliorating effects of the EACH on mouse cognitive impairment and hippocampal cell neurotoxicity are thought to be closely related to the physiological activity of its main component, KYNA.

As mentioned above, in order to study the therapeutic and alleviating efficacy of neurodegenerative diseases, including AD, it is a good method to explore the effects on related symptoms, including cognitive decline and neurotoxicity. Therefore, behavioral testing in an SCO-induced animal model similar to cholinergic cognitive impairment or a neurotoxicity model in hippocampal cells induced by oxidative stress is considered an appropriate research method. Evaluating the effects on mitochondrial dysfunction caused by excessive ROS accumulation, the efficacy of the activation of antioxidant mechanisms, including Nrf-2, and the efficacy of the TrkB/CREB mechanism related to hippocampal function are also considered promising research strategies for maintaining neuronal function. In this study, it was demonstrated that the EACH alleviates cholinergic cognitive impairment in a mouse model and effectively suppresses oxidative stress-induced neurotoxicity by suppressing ROS accumulation and mitochondrial dysfunction. Specifically, it was also demonstrated that this efficacy is exerted through the activation of the Nrf-2 antioxidant mechanism and the TrkB/CREB pathway. Since the research methodologies listed above are quite similar to the clinical symptoms of neurodegenerative diseases, the efficacy of the EACH proven in this study has an important meaning that it can be expected to be applied to clinical practice through additional research. EACH is a honey-derived substance used as a food that can be relatively safe among natural substances, so if cross-validation of its efficacy and securing of its safety are proven through subsequent research, it has sufficient value as a candidate for treatment or supplement for neurodegenerative diseases.

## 5. Conclusions

In conclusion, this study demonstrated that the EACH effectively alleviated SCO-induced cognitive impairment in mice and prevented glutamate-induced neuronal damage. Specifically, the EACH significantly reduced the escape distance and latency in the MWM test and significantly increased the step-through latency in the PA test, indicating that it effectively reversed the decrease in spatial memory function and working memory induced by SCO administration. In addition, the EACH effectively inhibited glutamate-induced neuronal cytotoxicity and signs of neuronal damage, such as LDH leakage and ROS overproduction, in HT22 hippocampal cells. Furthermore, the EACH preserved mitochondrial function by preventing MMP loss and effectively prevented HT22 apoptosis by regulating the expression of apoptosis-related proteins. These neuroprotective effects of the EACH may be due to the induction of antioxidant enzymes mediated by Nrf-2 and activation of the TrkB/CREB/BDNF pathway. Additionally, the cognitive improvement and neuroprotective effects of the EACH may be closely related to its main component, KYNA, which is known to exhibit neuroprotective effects and improve AD-like symptoms in mice. Overall, the EACH shows significant efficacy against parameters similar to the major clinical symptoms of neurodegenerative diseases, and as it is a substance derived from honey, a food material, it is believed that it can have sufficient potential value as a candidate for the treatment and supplement of neurodegenerative diseases through further research.

## Figures and Tables

**Table 1:** Primary and secondary antibodies used for Western blotting.

Antibody	Corporation	Product No.	RRID	Dilution Rate
Bcl-2	Cell Signaling	#3498	AB_1903907	1:1000
AIF	Cell Signaling	#4642	AB_2224542	1:1000
PARP	Cell Signaling	#9532	AB_659884	1:1000
β-actin	Cell Signaling	#4970	AB_2223172	1:1000
HO-1	Cell Signaling	#82206	AB_2799989	1:1000
NQO1	Santa Cruz	#SC-32793	AB_628036	1:1000
GCLC	Thermo Fisher	#PA5-87854	AB_2804457	1:1000
Nrf-2	Cell Signaling	#12721	AB_2715528	1:1000
Lamin B1	Cell Signaling	#13435	AB_2737428	1:1000
P-TrkB	Cell Signaling	#4621	AB_916186	1:1000
P-CREB	Cell Signaling	#9191	AB_331606	1:1000
BDNF	Cell Signaling	#47808	AB_2894709	1:1000
2nd anti-mouse	Cell Signaling	#7076	AB_330924	1:5000
2nd anti-rabbit	Cell Signaling	#7074	AB_2099233	1:5000

## Data Availability

The original contributions presented in this study are included in the article. Further inquiries can be directed to the corresponding authors.
